# Perceptions of Farm Animal Sentience and Suffering: Evidence from the BRIC Countries and the United States

**DOI:** 10.3390/ani12233416

**Published:** 2022-12-04

**Authors:** Fernando Mata, Bastian Jaeger, Ivo Domingues

**Affiliations:** 1 Centre for Research and Development in Agri-Food Systems and Sustainability, Instituto Politécnico de Viana do Castelo, 4900-367 Viana do Castelo, Portugal; 2 Department of Social Psychology, Tilburg University, 5037 AB Tilburg, The Netherlands; 3 Instituto de Ciências Sociais, Universidade do Minho, 4710-057 Braga, Portugal; 4 Centro de Estudos de Comunicação e Sociedade, Universidade do Minho, 4710-057 Braga, Portugal

**Keywords:** farm animal sentience, farm animal suffering, BRIC countries, USA, ethics of meat consumption, meat trading standards

## Abstract

**Simple Summary:**

The relations between farm animals and humans vary across countries and cultures. It was the aim of this study to understand the position of the population in the BRIC countries (Brazil, Russia, India, and China) and the USA. It was found that perceptions of farm animal sentience and suffering vary a lot with culture, country, gender, and age. This could have important consequences for the globalized trade of animal products does not find common grounds for standardization, and the risk of countries with more advanced animal welfare legislation imposing trade barriers increases. These trade barriers may be precepted as protectionism by exporting countries.

**Abstract:**

In this study, we examined how beliefs about farm animal sentience and their suffering vary across culture and demographic characteristics. A total of N = 5027) questionnaires were administered in Brazil, Russia, India, China, and the USA. Brazilians showed higher and Chinese lower levels of perceived animal sentience. In Russia and India, the perception of suffering and sentience increases with age, with similar levels to those observed in the USA. In all the countries, more people agreed than disagreed that animals are sentient. Men in India show higher levels of agreement with the relation between eating meat and animal suffering, followed by women in Brazil and China. Lower levels of agreement are observed in Americans and Chinese. Women show higher levels of compassion than men. In Russia, there is a slightly higher level of agreement between men and in the USA younger men agree more. Young American men show higher levels of agreement, while in India and China age has the opposite effect. For fair trading competition, it is important to standardize procedures and respect the demand for both animal protein and its ethical production. Overall, our results showed that perceptions of farm animal sentience and suffering vary substantially across countries and demographic groups. These differences could have important consequences for the perceived ethicality of meat production and consumption, and for global trade in animal products.

## 1. Introduction

Sustainability is a growing concern today. While aiming for agriculture production systems capable of feeding the world, we need to preserve the environment and use natural resources wisely. Under these circumstances, meat production and consumption patterns must also reach sustainable standards. Meat production is the main cause of greenhouse gas emissions and in the consumption of water. Together with sustainable standards, ethical standards are also demanded by today’s informed society.

Farm animal welfare (FAW) is part of those standards and is typically defined as “a potentially measurable quality of a living animal at a particular time” [1]. The first step for FAW is the ability to satisfy a basic need, often referred to as the five freedoms. The term “five freedoms” was coined in the UK by the Farm Animal Welfare Council, is now accepted worldwide, and refers to animals having [2]:

1. “Freedom from hunger and thirst, by ready access to water and a diet to maintain health and vigor.
2. Freedom from discomfort, by providing an appropriate environment.
3. Freedom from pain, injury, and disease, by prevention or rapid diagnosis and treatment.
4. Freedom to express normal behavior, by providing sufficient space, proper facilities, and appropriate company of the animal’s own kind.
5. Freedom from fear and distress, by ensuring conditions and treatment, which avoid mental suffering.”

From the perspective of the human relationship with the non-human animal, though, FAW as a construct must also include human obligations towards animals, often referred to as animal rights. It has been suggested that the five freedoms concept needs a broader interpretation [3] to include the recognition of animal sentience, defined as an animal “having the awareness and cognitive ability necessary to have feelings” [4]. Despite the existence of historical discourse about animals’ feelings, ranging from the classic Greek thinkers Hippocrates, and Pythagoras to Charles Darwin [5], most developments in the legal recognition of animal sentience have been made in recent years. European animals gained this official recognition in 1999 through the EU treaty of Amsterdam, which was later complemented with a protocol on their protection and welfare via the 2007 EU Treaty of Lisbon, adopted in 2009, and entering into force with the Directive 2010/63/EU. This event triggered a global reaction, in Western societies (e.g., Canada, Colombia, New Zealand, Switzerland, Turkey, Ukraine, USA) [6]. More recently, 180 countries adopted the OIE Global FAW Strategy 2017, including the recognition of animal sentience, and up to 46 countries supported the UN’s Universal Declaration on Animal Welfare [7].

The recognition that positive affective experiences are a good indicator of FAW is paramount in connecting animal sentience with more practical considerations of FAW [7]. In the present, anthropogenic suffering, or the acknowledgement of animal suffering caused by human actions, is seen as the major step towards an ethical relationship between humans and animals [8]. The role of humans in animal suffering is particularly apparent for farm animals that are kept with the explicit purpose of serving human needs [9].

Humans’ strong demand for animal products and widely held beliefs about the justifiability of meat consumption [10] makes complete independence from food of animal origin in the near future, unlikely. However, even in the short term, there is room for improvement in the sustainability of animal agriculture to preserve the environment, protect biodiversity, and feed the world’s growing population, while also working towards a more ethical treatment of farm animals, for example, by decreasing negative and promoting positive affective experiences in farm animals [11].

Yet, reducing the suffering of animals by changing the way in which humans treat them in food production may be a challenging task. Most people continue to consume meat even though they care about animals. This observation is often referred to as the meat paradox, defined as the cognitive dissonance or contradiction between the compassion felt by humans towards animals and the suffering imposed on them through the consumption of animal products [12]. Eating meat can become a particularly salient moral concern when people acknowledge that the animals, they are eating are sentient and capable of suffering. In fact, research has shown that people show increased moral concern for FAW when they are reminded that animals, such as humans, have the capacity to feel and think [13]. For example, Leach and colleagues [14] examined to what extent a variety of animal characteristics influence the perceived acceptability of eating the animal. People thought that it was less acceptable to eat animals that have the capacity to experience negative emotions. The relationship between perceived animal sentience and attitudes toward meat consumption seems to be bi-directional. That is, to avoid the negative cognitive consequences of holding dissonant attitudes (i.e., eating meat is permissible, but making animals suffer is not), people may categorize farm animals as less sentient and therefore less morally relevant when compared to companion animals [15]. Other strategies used by humans to deal with the cognitive dissonance resulting from meat consumption include endorsing speciesism, or the belief that humans are superior to other animals [16]; dissociating unpleasant ideas by referring to the bodies of sentient animals as ‘meat’ [15,17]; endorsing consumption as a social norm [18], and other strategies that are encapsulated in the 4N model: that meat is natural, necessary, normal, and nice [10]. Additionally, some people may acknowledge that animal farming can cause some animal suffering while asserting that farming procedures can be improved to minimize that suffering [12]. In short, beliefs about the sentience and suffering of farm animals are psychologically central to how people think about the ethicality of meat production and consumption.

Scholars have started to examine how perceptions of farm animal sentience and suffering differ across demographic groups. Some studies suggest that beliefs may differ between men and women [19,20]. One study [19] with students of different nationalities found higher levels of perceived animal suffering among women, but no difference with regard to perceived animal sentience. Cultural differences between countries were also identified with the authors reaching a generic conclusion that European and American students show stronger beliefs in animal suffering, than Asian students. Another study [20] reports stronger beliefs in animal sentience among female veterinary students, with similar tendencies across countries and cultures. In both studies individuals agreed on a hierarchy of the “capacity to feel” that places companion animals as more sentient, followed by farm animals and others.

The age effect has also been studied as a demographic variable capable of impacting perceived animal suffering and animal sentience. As well, the results obtained are mixed and dependent on the animal setting [21]. However, the authors cite a study [22] to justify a tendency to a natural shift in human perception as people become older. Older people tend to change priorities toward family needs and animals tend to be perceived as less important and seen more from a utilitarian point of view.

It is the aim of this study to examine beliefs about farm animal sentience and suffering of farm animals in the BRIC countries and the USA, and their variability across ages and gender. These beliefs are crucial for understanding how people think about the ethicality of meat production and consumption, which also has consequences for trade between countries. The global economy and trade are inevitable, but to achieve healthy trading competition it is important to standardize procedures and respect the demand for both animal protein and its ethical production. Nevertheless, religion, cultural differences, age, and gender influence public perceptions and awareness of humans’ relation with non-human animals [23,24]. The emerging economies of the countries known by the acronym BRIC (Brazil, Russia, India, and China) represent 40% plus of the world population and more than 50% of the world’s gross agricultural production in 2018 [25]. Thus, the present study explores beliefs that are central to how people think about the ethicality of meat production, a sector that is attracting increased attention in discussions about sustainability and climate change due to its important role as a greenhouse gas emitter, and the international trade of animal products. 

## 2. Materials and Methods

### 2.1. Source of Data

The data were collected by Faunalytics as part of an exploratory study on the attitudes and behaviors towards FAW among people in the BRIC countries and the United States (Anderson, 2018b). The data are available in the Open Science Framework repository. The data were collected by YouGov^®^ in May and June of 2018 from The BRIC countries and the USA. The sample of USA individuals is nationally representative, and it was a direct interview. The samples in Brazil, India, and Russia were collected around urban areas, also via direct interviews. The Chinese sample was collected through the internet. All the interviewees are above 18 years old. The individuals were randomly chosen from a YouGov^®^ panel of interviewee volunteers. The sample is stratified by gender and age. 

### 2.2. Data Collection

Data were collected online in the BRIC countries through questionnaires. These questionnaires were originally written in English and then translated into the local languages using a back-translation process in which the original translation from English to the local language was performed by one individual, while another translator who was blind to the original English version translated it back into English so that discrepancies could be caught. This procedure maximizes equivalence between countries and languages. Recommendations for keeping the wording as simple and direct as possible were also followed, using symmetrical response scales, and using both positively and negatively framed items.

The difficulties arriving from cross-cultural research are well studied. In order to overcome these we paid special attention to differences in interpretation, and in the use of response scales. Relatively to differences in interpretation the questions posed to the interviewees were checked against the good question wording [26]. As such the following was adopted to simplify without loss of meaning: short simple sentences, active voice, nouns instead of pronouns, and use of specific instead of general terminology. The following was avoided: metaphors, subjunctive, possessive form, vague words, and verbs suggesting different actions. An expert consultation phase prior to the finalization of the questionnaire was also included [27]. Relatively to differences in the use of response scales, we followed the recommendations to use symmetrical, bipolar response scales with a clear middle point [28].

### 2.3. Measures

Data included demographics (age, gender, country) and nine survey items. Two of these are part of the present study. Interviewees were asked several questions related to FAW, animal sentience, and their diet. In the present paper, we will investigate the responses related to the perceived sentience of animals (“animals used for food have approximately the same ability to feel pain and discomfort as humans”) and the perceived role of meat consumption in contributing to the suffering of animals (“eating meat directly contributes to the suffering of animals”). Participants responded to both items on a five-point Likert scale ranging from 1 (strongly disagree) to 5 (strongly agree).

### 2.4. Statistical Analysis

The original dataset was explored for the existence of outliers using Tukey’s method, with the production of boxplots. As a result, 28 observations were removed from the analysis. To investigate the agreement/disagreement with the statements listed in the previous section, each response scale was individually entered in a multinomial logistic regression as a function of the demographic variables age, gender, and country. The significance of the models was assessed with the −2 log likelihood chi-square test. The Akaike’s Information Criterion (AIC) is also shown for each of the models to be adjusted. The parameters were tested for significance using the Wald chi-square test. The statistical package used was the IBM Corp.^®^ SPSS^®^ Statistics, Armonk, NY, USA. Version: 28.0.1.1 (15). For the graphic construction, we used Microsoft^®^ Excel^®^ for Microsoft 365 MSO (version 2204 Build 16. 0. 15128. 20240) 64-bit.

The multinomial logistic models were fit to the data. The main effects were tested all together with a forward stepwise inclusion of interactions. The calculation of the probabilities (*P_i_*) of a national of a country to fall in a particular score while evaluating each of the statements is performed with the generic equation

(1) $$P_{i}=\frac{\exp\left(X_{i}\beta_{i}\right)}{1+\sum_{5}^{i=2}\exp\left(X_{i}\beta_{i}\right)}$$

where *P_i_* is the probability to score each of the “*i*” scores (2, 3, 4, 5). The equations’ parameters (*β_i_X_i_*) are arranged in a linear manner adopting the form

(2) $$\beta_{m}X_{m}+\beta_{1}X_{1}+\beta_{2}X_{2}+\beta_{3}X_{1}X_{m}+\beta_{4}X_{1}X_{2}+\beta_{5}X_{1}X_{2}+\beta_{6}X_{1}X_{2}X_{m}$$

where:

*β_m_* is the parameter associated with “Country” (Brazil, China, India, and Russia), being Xm the dummy associated with this parameter, taking the value one when the respective country is considered in the equation and zero otherwise. *β*_1_ is the parameter associated with the covariate “Age”, being *X*_1_ the age. *β*_2_ is the parameter associated with “Gender” (Man, Woman), being *X*_2_ the dummy associated and taking the value of one for men and zero for women. *β*_3_, *β*_4_, and *β*_5_ are the parameters associated with the two-way interaction terms (“Age × Country”, “Age × Gender”, and “Country × Gender”), and *β*_6_ is the parameter associated with the three-way interaction “Age × Gender × Country”.

Score 1 (strongly disagree) is used as reference and therefore for the calculation of P_1_ in equation (1) the numerator assumes the value 1.

The logistic coefficients (*β*) for each predictor variable for each alternative score of the statement are shown in the tables associated with the models representing the respective statements. The coefficients *β* are the expected amount of change in the logit for each one-unit change in the predictor. The logit is the odds of membership for each of the scores. The closer a logistic coefficient is to zero, the less influence the predictor has in predicting the logit. The exp(*β*) is the odds ratio associated with each predictor. Predictors increasing the logit have exp(*β*) > 1, those without effect on the logit have exp(*β*) = 1 and predictors deceasing the logit have exp(*β*) < 1. These correspond respectively to *β* coefficients above, equal, or below zero.

The following sections address each of the adjusted models for the different statements, showing the degree of adjustment, the parameter estimates, and the graphic representation and interpretation.

### 2.5. Analytical Procedure 

After adjusting the models with the five categories of the Likert scale, to facilitate the interpretation of the results, and construct simpler graphs, it was decided to aggregate the categories 1 (strongly disagree) with 2 (disagree) and the categories 4 (agree) with 5 (strongly agree). Three new main categories were therefore created: disagree, neutral, and agree. These are the categories herein represented graphically and subject to result interpretation and discussion.

## 3. Results

### 3.1. Descriptive Statistics

**Figure 1:**
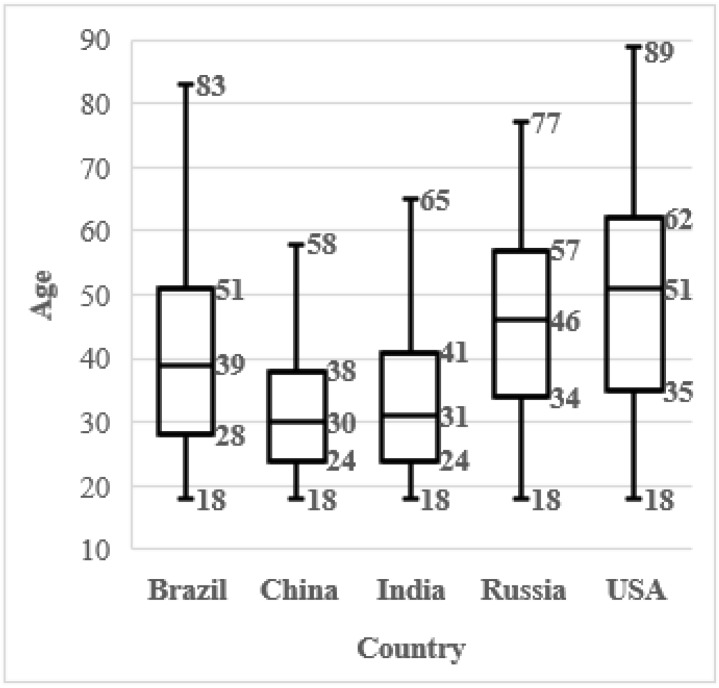
Sampled variables distribution. Ages within countries.

**Figure 2:**
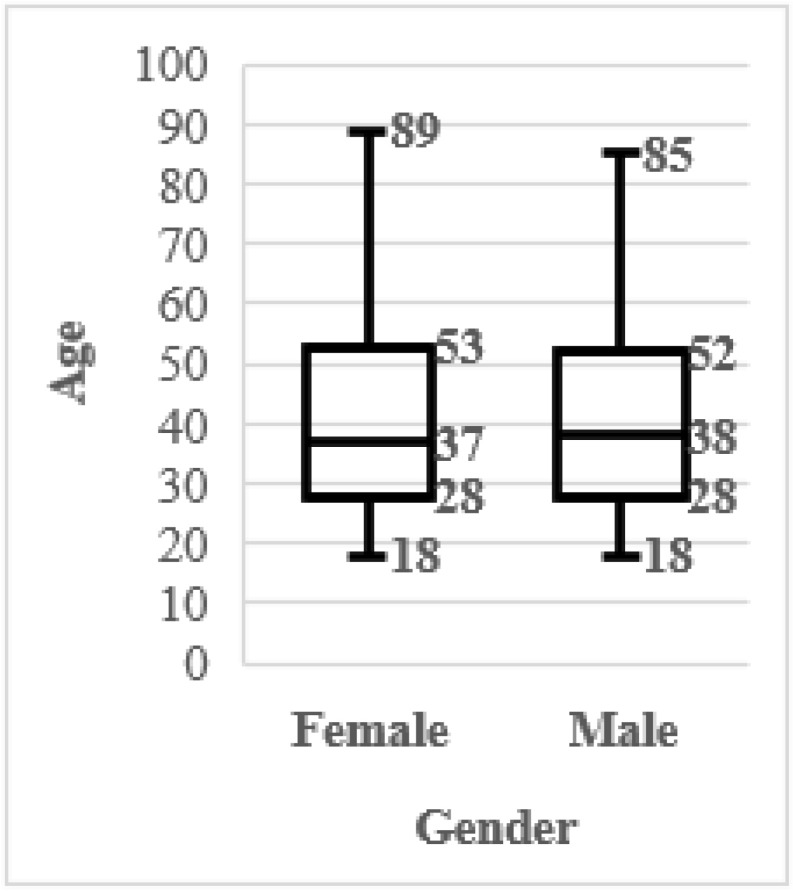
Sampled variables distribution. Ages within gender.

**Figure 3:**
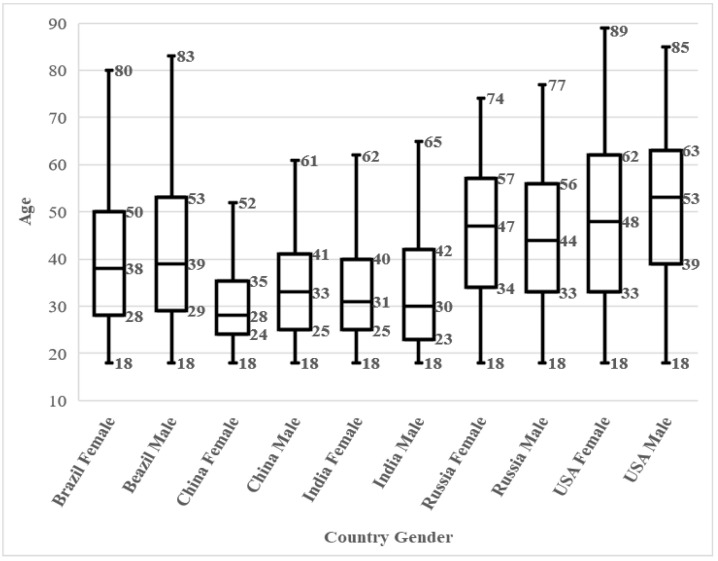
Sampled variables distribution. Ages within gender and countries.

A total of N = 5172 individuals were entered in the analysis (Brazil n = 1027, China n = 966, India n =1004, Russia n = 1002, USA n = 1173), including n = 2586 men and n = 2586 women. The age distribution within countries is shown in the boxplots in Figure 1. The age distribution for each gender is shown in boxplots in Figure 2. As can be observed data is well balanced for gender, while for age it is slightly skewed towards younger ages in China and India. When considering all the three independent variables together we can observe a slight skewing towards older ages in Chinese men, while for other countries gender is well balanced (Figure 3).

### 3.2. Fitted Models

#### 3.2.1. Perceived Animal Sentience

**Figure 4:**
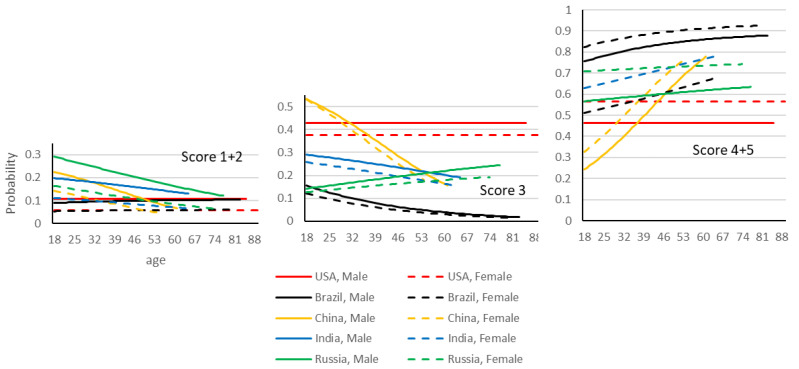
Graphical representation of the multinomial logistic model fitted to the data. The scores 1, 2 and 4, 5 are aggregated. Probabilities associated with disagreement scores (1 + 2), neither agree nor disagree score (3), and agreement scores (4 + 5) given to the statement “*animals used for food have approximately the same ability to feel pain and discomfort as humans*”.

The model examining the perceived sentience of animals was significant (*p* < 0.001), −2 log likelihood 5070, chi-square (859, 40df), and AIC 5158. The parameters found to be significant were (−2 log likelihood, chi-square, df, *p*-value): “country” (5176, 106, 16, *p* < 0.001), “gender” (5146, 75, 4, *p* < 0.001), and the interaction “age x country” (5141, 71, 20, *p* < 0.001). The percentage of respondents per score and country are given in Table 1. The description of the significant parameters of the model are given in Table 2. Figure 4 is the graphical representation of the model.

In general Brazilian participants showed stronger beliefs in animal sentience than other nationalities in the study. Chinese participants showed the lowest belief in animal sentience, and this was particularly true for Chinese participants below the age of 45. Note that the Chinese sample was on average much younger compared to other countries which may have impacted the overall results. Older participants in Russia and India had indicated stronger beliefs in animal sentience than younger participants in these countries. Both have similar levels of perception to those in the USA. China has the highest levels of neutral responses. Women showed stronger beliefs in animal sentience than men across the different countries. The levels of disagreement are all bellow a probability of 0.3 in the different countries, but men from Russia, India and China show the higher levels. Disagreement decreased with age in China, India, and Russia. All the countries showed a higher prevalence of participants who do (vs. do not) believe in animal sentience. 

#### 3.2.2. Perceived Animal Suffering

**Figure 5:**
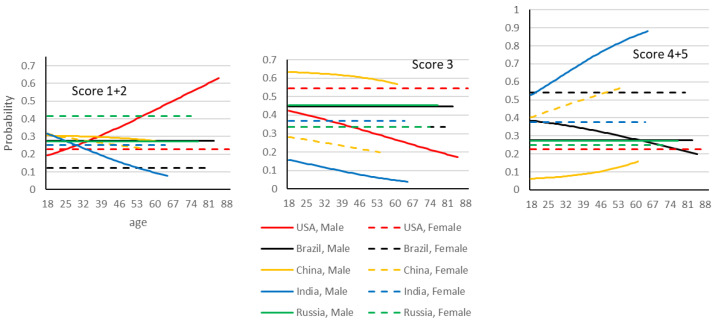
Graphical representation of the multinomial logistic model fitted to the data. The scores 1, 2 and 4, 5 are aggregated. Probabilities associated with disagreement scores (1 + 2), neither agree nor disagree score (3), and agreement scores (4 + 5) given to the statement “*eating meat directly contributes to the suffering of animals*”.

For this statement, the model is significant (*p* < 0.001), −2 log likelihood 5648, chi-square (1500, 80df), and AIC 5808. The parameters found to be significant were the interactions (−2 log likelihood, chi-square, degrees of freedom, *p*-value): “country x age” (5885, 238, 40df, *p* < 0.001), and “gender x age x country” (5748, 100, 40df, *p* < 0.001). The percentage of respondents per score and country is given in Table 3. The description of the significant parameters of the model is given in Table 4. Figure 5 is the graphical representation of the model.

Men in India showed stronger beliefs in the relation between eating meat and animal suffering than women, followed by women in Brazil and China. Lower levels of agreement are observed in Americans and Chinese, especially younger Chinese men. Americans and Chinese also indicated neutral responses more often. Women reported stronger beliefs in animal suffering than men, and the difference is especially large in China, Brazil, and among older individuals in India. Stronger beliefs were also observed in Russia among men and in the USA among younger men. The decline with age in the USA stands in opposition to Indian men, and Chinese individuals, where the prevalence of belief in animal suffering increases with age.

Neutral responses are especially common among Chinese men (decreasing with age) and American women. Neutrality decreases with age in Chinese participants, and in American men and Indian men.

Disagreement with the relationship between eating meat and animal suffering is especially high in older American men and Russian women. It is lower for Brazilian women and older Indian men. 

## 4. Discussion

### 4.1. Country Effects

China is a global leading producer of meat (poultry, pork, dairy, and wildlife products) [29]. However, the stark increase in production that took place come at the expense of FAW [30,31]. Farm animal welfare science originated in Western societies and was introduced in China only in the 1990s [32]. There is however a growing awareness among the Chinese scientific community and in the population in general, due to the influence of Western culture and the awareness of the relationship between FAW and meat quality [33,34]. Despite this growing awareness, FAW issues are still enormous, including a lack of legislation and poor slaughter procedures [30,35]. When considering the countries in the present study, China has the overall lowest animal protection index.

The major Chinese religions (Buddhism, Confucianism, and Taoism) shape cultural attitudes toward animals in the country, more than any Western philosophy [36]. Buddhism, however, shares similar values with the animal liberation theories of the West, where consideration is given to every living being, along with the right to a life free from suffering [37]. Confucianism and Taoism give the same consideration to animals as to humans [38]. Nevertheless, popular practices of animal trading and consumption still cause a lot of animal suffering [39]. Despite prescribing respect for non-human animals, Confucianism places non-human animals in an inferior status in relation to humans and advocates for rituals and offers involving the consumption of animals [40]. 

Chinese youth underwent an important shift in market economy, urbanization, and globalization when compared with the previous generation [41]. The globalization of Chinese society brought cultural and ideological conflicts with the Western countries and the younger generations entered into a societal change [33]. On the other hand, the increasing competition pressure is also shaping the younger generations in China, widening the generation gap [41]. A survey of Chinese college students showed that the majority recognize animal sentience [42], with stronger recognition in rural areas. 

Considering animal sentience, in this study, Chinese participants showed a response pattern that completely differentiates them from the other nationalities. A positive age effect is very pronounced both in men and women, with the acknowledgement of animal sentience growing exponentially approximately up to the age of 50, reaching extremely prominent levels at this stage. A possible explanation for this may be the higher degree of commitment to traditional religious practices, and the rural origin of much older Chinese, which typically exhibit a stronger endorsement of animal sentience [32]. This can also explain the higher levels of neutral responses, especially in younger ages. Nevertheless, perceptions of animal sentience are lower than in any other countries in the study, which is partly due to the exceptionally low levels of perceived sentience among younger Chinese participants.

With regard to belief in farm animal suffering, most Chinese men took a neutral position, while the agreement was higher among women. Again, a positive effect of age was apparent. Levels of disagreement are similar to other countries in the study.

In India, cruelty against animals has always been under philosophic religious scrutiny due to beliefs in non-violence purity, and reincarnation [43]. Cattle have an important religious role for Hindu people being widely venerated and typically only slaughtered by individuals from lower casts [44].

The cultural differences between Indians and Western cultural attitudes towards animals have been evident from the moment Europeans arrived and settled in India. The condemnation of some native practices prompted the British to pass legislation against animal cruelty introduced in Bengal in 1869. However, the colonial practices of slaughtering cattle to feed armies or hunting expeditions were perceived as contradictory and have not been well understood by the locals [45].

Indians were perceived to be vegetarians, but meat has always been a central part of their diet. However, the colonizers’ diet included much more meat, which led to the perception of vegetarianism by comparison [46,47]. Nevertheless, the nationalism that led to Indian independence was followed by legislation prohibiting the slaughtering of the sacred cow [48]. More recently the ban on beef in India raised issues with the Christian, Muslim, and Dalit (lower caste) communities, who accused the government of forgetting the multicultural heritage of India, favoring Hindus [49].

Indian society experienced significant changes in the 1990s, with a new middle class being able to afford an improved diet to include meat [50]. Nowadays about 80% of the population is Hindu and 70% of the population eats meat [51]. Nevertheless, despite recent increases in meat consumption, India remains one of the nations in the world with the lowest meat consumption per capita [52]. Vegetarians are especially motivated by the moral concern related to farming and slaughtering [46].

Indians showed high levels of perceived animal sentience, especially among older participants. Older individuals are more religious and therefore tend to be more aligned with the vegetarianism tradition of the Hindu culture [46]. This age effect is even more pronounced when examining agreement with the statement that animal farming causes suffering. The rate of neutral respondents is similar to other countries.

Brazil is an important world meat producer and holds a tradition of meat consumption, with the livestock sector of the country accounting for 8.7% of the GDP in 2019 [53]. As an emerging economy, Brazil is still experiencing changing diets including the inclusion of more animal protein, and therefore meat consumption is still expected to increase [54].

Brazil is a multicultural nation; however, Christianity is predominant. As such there are no meat-eating restrictions. Certain Afro-Brazilian religions, predominantly Candomblé, gained the status of national tradition, without ethnical or social boundaries, and are even incorporated by the local Christians [55]. These traditions include rituals of animal blood sacrifices offered to the orisha (gods). These sacrifices are taken in festivities which include eating the animals, and may include, goats, chicken, ducks, pigs, sheep, other birds, turtles, cavy, and snails [56].

The Brazilians showed the strongest belief in animal sentience, higher than all other countries that were part of this study, which tallies the results obtained in a study comparing Brazilian and French [57]. Important levels of animal sentience perceptions among sheep farmers in Brazil have also been reported [58]. In the current study, the perceived suffering of animals due to meat consumption was especially acknowledged by Brazilian women.

Meat consumption has also been growing in Russia. Recently during the embargo imposed by the EU in 2014, the government has implemented policies to attain self-sufficiency in meat production and to reach higher levels of animal protein consumption in poorer segments of society [59]. Indigenous populations of Siberia still rely on the wild meat of ungulates, but consumption of other animals such as bears is also common [60]. Russia has an Orthodox Christian heritage which translates to lack of restrictions on eating meat. Russians have a strong preference for meat and meat consumption can be expected to grow if affordable [61]. 

Russian participants showed similar beliefs in animal sentience and compared to participants of other nationalities in this study. Russian women showed particularly higher levels of disagreement with the statement relating meat consumption and animal suffering. 

The USA is among the top consumers of meat in the world [52]. Meat-based diets, especially red and processed meat are common in the USA, which is positively correlated with obesity (Rouhani et al., 2014). The USA tops the list of countries of meat production and consumption per capita [52] and is among the countries with higher obesity rates [62].

Despite the health effects and the effects on the sustainability of the environment, consumerism in the USA is still finding room for increasing consumption. The “growth in advocacy for animal rights and environmental sustainability often cannot match the powerful lobby of meat producers that exerts influence on politicians (e.g., via donations to certain candidates) and the public (e.g., via TV commercials). In a study [63] about the influence of the media on meat consumption in the USA, it was found that individuals more exposed to media content, dissociate more often meat from its animal origin and eat more meat. Examples of dissociation include the use of terms such as drumsticks and buffalo wings to dissociate from chicken; the use of the words “pork” or “beef” to dissociate from pigs and cattle; sculptured made nuggets to appeal to children and dissociate the product from chicken, and processed meat such as cheeseburgers. The livestock industry in the USA is the most productive, per capita, in the world.

USA participants showed weaker beliefs in animal sentience in comparison to the BRIC countries. Only China showed lower levels of perceived animal sentience. While the prevalence of neutral responses was especially high in China, disagreement with the statement that animals can feel pain and discomfort was higher in the USA.

Regarding the acknowledgement of farm animal suffering caused by meat consumption, scores in the USA were comparable to scores in the BRIC countries. However, in older men acknowledgment was particularly low compared to the other countries. In a recent study in the USA, about 30% of the population was reported to acknowledge that farm animal suffering should be given the same consideration as human suffering [63].

### 4.2. Age Effects

Younger people consume more meat in the countries that were part of this study: USA [64], Brazil [65], China [66], and India [67], but not in Russia [68]. Older individuals consume less meat which correlates positively with a lower need to justify the morals of farming and fishing [69]. Older people more frequently justify the morals of eating meat with the 4 Ns (natural, necessary, normal, or nice) [10]. Older people also score lower on perceived animal sentience [70]. However contradictory results are also found in research relating age to meat eating and its moralities [71].

Perceptions of animal sentience showed a positive correlation with age. Perceptions of animal suffering also increased with age among Chinese individuals and Indian men, while the opposite is observed for men in the USA. The other pairs’ country/gender show no age effects.

### 4.3. Gender Effects

Men and women have been shown to behave differently in relation to meat consumption and its perceived morality. Men consume proportionally more meat than women, and meat consumption is stereotypically linked to power, dominance, and masculinity, the roles traditionally embraced by men [72]. Men also show lower levels of perceived animal sentience, and more frequently deny farm animal suffering [72]. Men also use the 4Ns strategy more often to justify eating meat [10]. Women on the other hand have been shown to be more concerned about animal rights and ethics [73]. Women present stronger critical attitudes towards meat eating, disgust, and repulsion [74]. The suffering of animals is a stronger concern for women who are also more likely to turn vegetarian [75]. Gender attitudes can also influence meat-eating attitudes, and societies with higher levels of hostile sexism and lower levels of women empowerment correlate with greater gender differences in meat-eating attitudes [76]. 

The consumption of meat by men is higher than that of women in all the countries of the present study: USA [64], Brazil [77], China [78], India [50], and Russia [61]. We found that women reported stronger beliefs in animal sentience across all the countries in this study. Regarding the perceived suffering of farm animals, the gender results are mixed. Women acknowledged farm animal suffering more than men in Brazil, China, and from the age of 80 in the USA, but not in other countries. Particularly Russia has a prominent level of women that do not acknowledge the suffering of farm animals.

### 4.4. The Ethicalities of Meat Consumption

Studies show us that meat consumption raises a diversity of ethical questions among consumers [79,80]. Namely, meat provides essential nutrients, such as amino acids, energy, and micronutrients, and gives sensorial pleasure due to unique organoleptic properties [10]. However, if consumed in excess is also a cause health problems, such as cardiovascular diseases and colorectal cancer [79]. Meat consumption is legitimated by cultural tradition but also raises sustainability issues due to high levels of water consumption and greenhouse gas emissions [79,80].

Due to this dichotomy between benefits and costs, meat consumption has become a topic of ideological battles and paradoxical tensions. Some scholars argue that meat consumption is “unhealthy, unsustainable, and unethical”. These paradoxical tensions can be seen as the “two sides of the same coin” as the need for meat production to satisfy human needs brings the referred issues [81].

Some of the solutions being proposed include the reduction of meat consumption and the diversification of protein sources from the non-animal origin [82]. However, at the same time, it is proposed that meat should not be fully replaced [81]. Therefore, meat consumption is characterized by this systemic paradoxical tension for which, and with the actual food technology state of the art, an optimal solution was still not found.

The actuality shows that most people agree that doing harm to animals is wrong [83] and that sentient beings should be treated morally [84]. In the present study, we have found high levels of agreement with both the suffering and the sentience question, which reveals the ethical consideration that people give to animals. This stands in contrast with the widespread harm still inflicted on animals in society.

The present study demonstrates the diversity of beliefs in farm animal sentience and anthropogenic animal suffering. Although we examined variation across gender, nationality, and age, we acknowledge that other factors are also a source of variation. Other variables not included in this study may impact the perceived animal suffering and animal sentience, and these include personality, religious and/or political stance, and ownership of companion animals [23].

### 4.5. Limitations and Future Research

The current analysis was quantitative and descriptive in nature, examining relationships between central beliefs about animals (animal sentience and suffering of farm animals) and demographic characteristics (country, gender, and age)). The results obtained to allow a snapshot of the situation, but do not necessarily fully explain the reality. Future research may investigate other type of theoretical models from a cultural perspective. Studies focused on the comparison of societal diversity have shown that culture has an enormous influence on people’s behaviors, and therefore social behaviors vary immensely [85]. 

Culture is defined [86] as “the collective programming of the mind which distinguishes the members of one human group from another”. This does not however mean that every member of a particular society shares the same programming; the national culture is only the dominant mental program [86]. National culture naturally varies accordingly to gender and age; being masculinity, femininity, and age determined by national characteristics, therefore subject to analysis [86].

Through the definition of culture, we can immediately infer limitations in this study. First of all, these type of studies are hard to implement; secondly, once they are directed to a specific societal subject, reduces the plausibility of a broader national culture; and third, it involves a society confined to a geography, eventually hiding the impact of variables such as religious differences mainly in nations such as China and India, but also in the others, and the original cultures of immigrants, mainly in the case of the USA and Brazil.

Research-based on the theory of lifestyles may be useful in the phenomenon herein studied and analyzed. This theory is based on habits, which is seen as the principle in the genesis of the portrayal of intrinsic and relational characteristics in a unitary lifestyle; in other words, a homogeneous set of people’s choices, beliefs, and practices, allowing the explanation of practices and judgments in a distinct circle [87]. Therefore, the selection or creation of lifestyles is influenced by group and peer pressures and the visibility of their role models, together with the socio-economic variables. As a result, lifestyles have been used in a theoretical model for analysis of reality, and in the support of segmentation strategies to study consumers’ behaviors [88,89,90]. Hence, the adoption of a theoretical framework based on the lifestyle’s theory could improve the scientific capacity to analyze the judgements and practices of consumers related to the ontological nature of animals and the consumers’ impact on its suffering, as well as perception of animal sentience.

Moreover, the sampling strategy implemented by YouGov^®^ does not fully represent a pure random sample of the vast population in the studied countries. Despites the large sampling effort of around one thousand interviewees per country. Despites all the efforts to implement a valid construct of the questions posed to the interviewees, the existence of cross-cultural differences may impact negatively the evaluation of the sentience and suffering concepts across nations.

### 4.6. Implications

The results of this study contribute knowledge to stakeholders, policymakers and traders. These may be used by these supply chain players to program and overcome problems related to the global FAW standards. For fair trading competition it is important to standardize procedures and respect the demand for both animal protein and the ethics of its production. If international common grounds for standardization are not found, the risk of countries with more advanced animal welfare legislation imposing trade barriers increases. These trade barriers may be perceived as protectionism by exporting countries.

## 5. Conclusions

Farm animal welfare is growing in the political agenda in many countries, and the global trade of animal products is a reality. However, FAW, sentience, suffering, and the ethicalities of meat consumption vary between cultures, gender, and across ages. 

In Russia and India, perception of FAW suffering and FAW sentience increases with age, with similar levels to those observed for the USA. Women have higher levels of perceived animal sentience and suffering in all the countries included in this study. Also, in all the countries, more people agreed than disagreed that animals are sentient. Men in India show higher levels of agreement with the relation between eating meat and animal suffering, followed by women in Brazil and China. Lower levels of agreement are observed in Americans and Chinese. In Russia there is a slightly higher level of perceived FAW suffering between men and between younger Americans. In India and China age has the opposite effect.

The paradoxicality of meat consumption remains an ethical concern promoting the debate of sustainability.

## Figures and Tables

**Table 1:** Percentage of respondents to the question in each of the countries in each of the categories disagree, neutral and agree.

Question	Brazil	Russia	India	China	USA
Animals used for food have approximately the same ability to feel pain and discomfort as humans					
Disagree (%)	7	14	14	12	10
Neutral (%)	15	24	18	50	28
Agree (%)	79	63	67	37	62

**Table 2:** Parameters of the multinomial logistic model fitted to the data. The ordinal dependent variable is the statement “*animals used for food have approximately the same ability to feel pain and discomfort as humans*,” modeled as a function of the independent variables “Country”, Gender” and “Age” together with the interactions between these. Only significant (*p* < 0.05) parameters are shown. Score 1 is used as reference in the model.

Score	Parameter		*β*	exp(*β*)
2	Country	Brazil	2.336 **	10.345
	Gender	Male	−0.407 *	0.665
3	Country	Brazil	3.902 ***	49.518
		India	1.533 ***	4.633
		China	2.016 *	7.509
		USA	2.541 ***	12.692
	Gender	Male	−0.638 ***	0.529
	Country × Age	Brazil, Age	−0.036 *	0.965
		Russia, Age	0.024 *	1.024
4	Country	Brazil	4.432 **	84.058
		Russia	1.285 **	3.614
		India	1.738 **	5.684
		USA	2.123 **	8.359
	Gender	Male	−0.883 ***	0.414
	Country × Age	China, Age	0.062 *	1.064
5	Country	Brazil	4.508 *	90.744
		Russia	1.177 *	3.243
		India	1.276 **	3.581
		USA	2.376 *	8.435
	Gender	Male	−1.050 ***	0.350
	Country × Age	China, Age	0.104 ***	1.110
		India, Age	0.025 *	0.021
		Russia, Age	0.024 *	1.024

Note: * *p* < 0.05, ** *p* < 0.01, *** *p* < 0.001.

**Table 3:** Percentage of respondents to the question in each of the countries in each of the categories disagree, neutral, and agree.

	Brazil	Russia	India	China	USA
Eating meat directly contributes to the suffering of animals					
Disagree (%)	27	32	21	23	33
Neutral (%)	28	33	28	45	36
Agree (%)	45	35	51	31	30

**Table 4:** Parameters of the multinomial logistic model fitted to the data. The scores given to the statement “*eating meat directly contributes to the suffering of animals,*” used as the dependent variable, are modeled function of the independent variables “Country”, Gender” and “Age” together with the interactions between these. Only significant (*p* < 0.05) parameters are shown. Score 1 is used as a reference in the model.

Score	Parameter		*β*	exp(*β*)
2	Country × Gender	China, Male	1.686 *	5.396
		India, Female	1.851 *	6.365
		Russia, Female	2.308 ***	10.050
	Country × Gender × Age	China, Female, Age	0.150 *	1.162
3	Country × Gender	Brazil, Male	1.174 *	3.235
		Brazil, Female	1.693 **	5.435
		China, Male	2.586 ***	13.275
		India, Female	2.379 ***	10.796
		Russia, Male	1200 *	3.322
		Russia, Female	2.191 **	8.947
		USA, Male	2.038 ***	7.673
		USA, Female	1.571 ***	4.809
	Country × Gender × Age	China, Female, Age	0.147 *	1.159
		USA, Male, Age	−0.031 ***	0.970
4	Country × Gender	Brazil, Female	1.698 **	5.464
		India, Female	2.387 ***	10.878
		Russia, Female	1.730 *	5.643
		USA, Male	1.288 **	3.625
	Country × Gender × Age	China, Female, Age	0.166 **	1.181
		USA, Male, Age	−0.025 **	0.975
5	Country × Gender	Brazil, Female	1.193 *	3.298
		China, Male	−2.126 *	0.119
		China, Female	−3.934 *	0.20
		India, Female	−2.057 **	7.823
		USA, Male	1.097 *	2.996
	Country × Gender × Age	China, Male, Age	0.051 *	1.053
		China, Female, Age	0.163 *	1.178
		India, Male, Age	0.047 **	1.048
		USA, Male, Age	−0.032 ***	0.968

Note: * *p* < 0.05, ** *p* < 0.01, *** *p* < 0.001.

## Data Availability

The data used in this study is available at the Open Science Framework repository: Anderson, J., 2018b. BRIC dataset.sav [data set]. Faunalytics page in the Open Science Framework repository. https://osf.io/stbxc/ (accessed on 12 March 2022).
